# Undeca­carbonyl-1κ^3^ *C*,2κ^4^ *C*,3κ^4^ *C*-[tris­(4-methyl­phen­yl)arsine-1κ*As*]-*triangulo*-triruthenium(0)

**DOI:** 10.1107/S1600536809046935

**Published:** 2009-11-21

**Authors:** Omar bin Shawkataly, Imthyaz Ahmed Khan, Chin Sing Yeap, Hoong-Kun Fun

**Affiliations:** a Chemical Sciences Programme, School of Distance Education, Universiti Sains Malaysia, 11800 USM, Penang, Malaysia; b X-ray Crystallography Unit, School of Physics, Universiti Sains Malaysia, 11800 USM, Penang, Malaysia

## Abstract

In the title *triangulo*-triruthenium compound, [Ru_3_(C_21_H_21_As)(CO)_11_], one equatorial carbonyl group has been substituted by the monodentate arsine ligand, leaving one equatorial and two axial carbonyl substituents on the Ru atom. The remaining two Ru atoms each carry two equatorial and two axial terminal carbonyl ligands. The three arsine-substituted phenyl rings make dihedral angles of 73.2 (2), 71.0 (2) and 75.3 (2)° with each other. In the crystal packing, mol­ecules are stacked down the *b* axis and each mol­ecule is stabilized by an intra­molecular C—H⋯O hydrogen bond.

## Related literature

For general background to *triangulo*-triruthenium derivatives, see: Bruce *et al.* (1985 ▶, 1988*a* ▶,*b* ▶); Shawkataly *et al.* (1998 ▶, 2004 ▶). For related structures, see: Shawkataly *et al.* (2006 ▶, 2009*a* ▶,*b* ▶). For the stability of the temperature controller used for the data collection, see: Cosier & Glazer (1986 ▶).
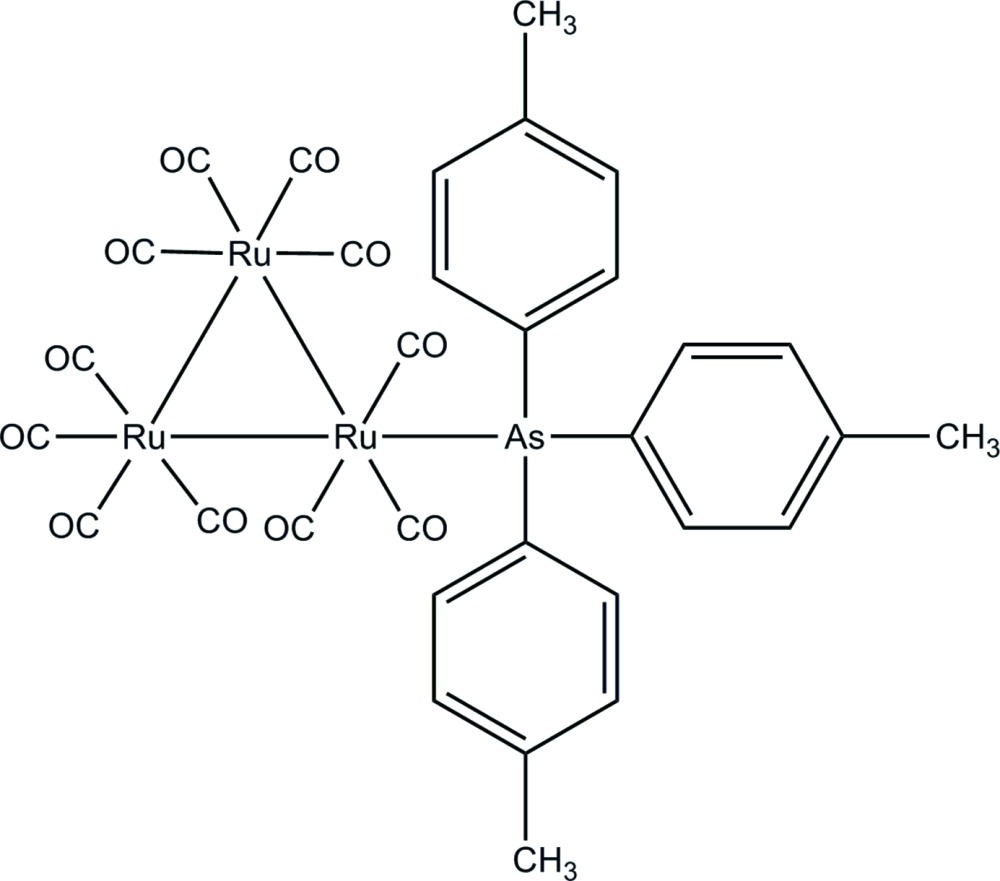


## Experimental

### 

#### Crystal data

[Ru_3_(C_21_H_21_As)(CO)_11_]
*M* *_r_* = 959.62
Triclinic, 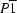

*a* = 10.5081 (3) Å
*b* = 11.2922 (3) Å
*c* = 14.4625 (4) Å
α = 98.326 (1)°
β = 94.056 (2)°
γ = 98.923 (1)°
*V* = 1669.83 (8) Å^3^
*Z* = 2
Mo *K*α radiation
μ = 2.38 mm^−1^
*T* = 100 K
0.33 × 0.16 × 0.08 mm

#### Data collection

Bruker SMART APEXII CCD area-detector diffractometer
Absorption correction: multi-scan (**SADABS**; Bruker, 2005 ▶) *T*_min_ = 0.511, *T*_max_ = 0.838
39493 measured reflections
9594 independent reflections
7073 reflections with *I* > 2σ(*I*)
*R*_int_ = 0.051

#### Refinement

*R*[*F* ^2^ > 2σ(*F* ^2^)] = 0.048
*wR*(*F* ^2^) = 0.133
*S* = 1.06
9594 reflections
427 parameters
H-atom parameters constrained
Δρ_max_ = 2.32 e Å^−3^
Δρ_min_ = −1.20 e Å^−3^

### 

Data collection: *APEX2* (Bruker, 2005 ▶); cell refinement: *SAINT* (Bruker, 2005 ▶); data reduction: *SAINT*; program(s) used to solve structure: *SHELXTL* (Sheldrick, 2008 ▶); program(s) used to refine structure: *SHELXTL*; molecular graphics: *SHELXTL* software used to prepare material for publication: *SHELXTL* and *PLATON* (Spek, 2009 ▶).

## Supplementary Material

Crystal structure: contains datablocks global, I. DOI: 10.1107/S1600536809046935/sj2671sup1.cif

Structure factors: contains datablocks I. DOI: 10.1107/S1600536809046935/sj2671Isup2.hkl

Additional supplementary materials:  crystallographic information; 3D view; checkCIF report

## Figures and Tables

**Table 1:** Hydrogen-bond geometry (Å, °)

*D*—H⋯*A*	*D*—H	H⋯*A*	*D*⋯*A*	*D*—H⋯*A*
C14—H14*A*⋯O3	0.93	2.48	3.385 (5)	165

**Fig. 1.**
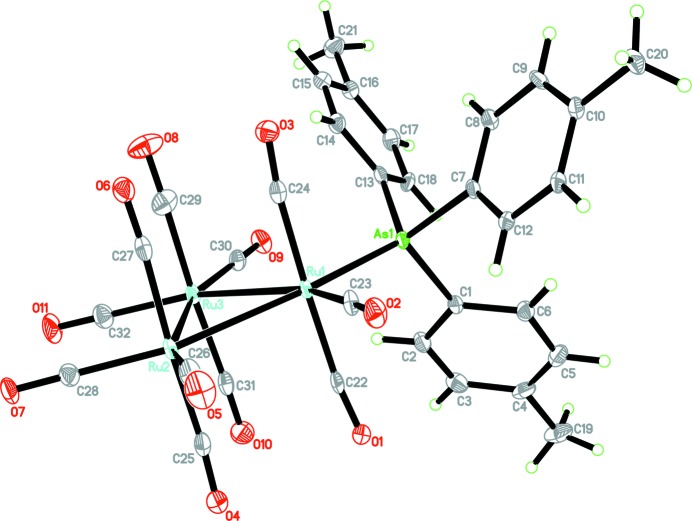
The molecular structure of the title compound with 30% probability ellipsoids for non-H atoms.

**Fig. 2.**
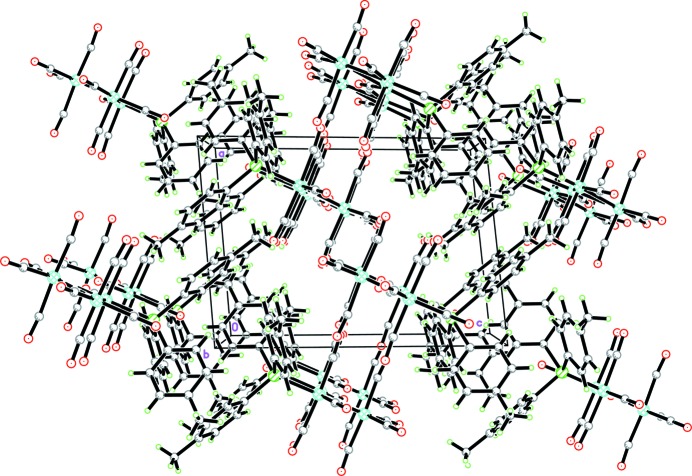
The crystal packing of the title compound, viewed down the b axis, showing the molecules stacked down b axis.

## supplementary crystallographic information

### Comment

Tri-angulotriruthenium clusters are known for their interesting structural variations and related catalytic activity. A large number of substituted derivatives, Ru_3_(CO)_12-n_*L*_n_ (*L*=15 group ligand) have been reported (Bruce *et al.*, 1988*a*,*b*; Bruce *et al.*, 1985). In continuation of our interest in the substituted clusters (Shawkataly, Ramalingam *et al.*, 1998, 2004; Shawkataly *et al.*, 2005, 2009*a*) we report here the synthesis and structure of Ru_3_(CO)_11_(As(4-CH_3_C_6_H_4_)_3_).

The bond lengths and angles of title compound (Fig. 1) are comparable to those found in related structures (Shawkataly *et al.*, 2006, 2009*a*,*b*). The tri-angulotriruthenium is bonded to a monodentate arsine ligand. The arsine ligand is equatorial with respect to the Ru_3_ triangle. Additionally, the Ru2 and Ru3 atoms each carry two equatorial and two axial terminal carbonyl ligands except for arsine-bonded Ru1 atom which binds one equatorial and two axial terminal carbonyl ligands. The three arsine-substituted benzene rings make dihedral angles of 73.2 (2), 71.0 (2) and 75.3 (2)° with each other.

In the crystal packing, the molecules are stacked down *b* axis (Fig. 2) and each molecule is stabilized by an intramolecular C14—H14A···O3 hydrogen bond (Table 1).

### Experimental

The reactions were conducted under an atmosphere of high purity nitrogen using standard Schlenk techniques and tetrahydrofuran (THF) was dried over sodium metal. Tris(4-methylphenyl)arsine (Maybridge) is used as received. Ru_3_(CO)_12_ (100 mg, 0.16 mmol) and tris(4-methylphenyl)arsine (56 mg, 0.16 mmol) were stirred in THF (25 ml) under nitrogen. About 0.2 ml of diphenylketyl radical anion initiator was introduced into the reaction mixture under a current of nitrogen. The reaction mixture turned intense red. After 10 minutes of stirring the solvent was removed under vacuum. The reaction mixture was separated by TLC (acetone:hexane, 10:90); three bands appeared. The major band (red) *R*_f_ = 0.78 yielded the title compound which was crystallized from CH_2_Cl_2_—CH_3_OH, yield = 100 mg, 57%, m.p. 137 °C. IR(cyclohexane).ν(CO) 2173 s, 2045 s, 2030 s, 1988 s, cm^-1^. ^1^H NMR (CDCl_3_) δ 7.29 (6 H, d, J = 7.98 Hz), δ 7.24 (6 H, d, J = 7.86 Hz), δ 2.40 (s, 9 H, 3CH_3_).

### Refinement

All hydrogen atoms were positioned geometrically and refined using a riding model with C—H = 0.93–0.96 Å and *U*_iso_(H) = 1.2 or 1.5 *U*_eq_(C). A rotating group model was used for the methyl groups.

### Crystal data

**Table:**

[Ru_3_(C_21_H_21_As)(CO)_11_]	*Z* = 2
*M**_r_* = 959.62	*F*(000) = 932
Triclinic, *P*1	*D*_x_ = 1.909 Mg m^−^^3^
Hall symbol: -P 1	Mo *K*α radiation, λ = 0.71073 Å
*a* = 10.5081 (3) Å	Cell parameters from 9957 reflections
*b* = 11.2922 (3) Å	θ = 2.5–29.8°
*c* = 14.4625 (4) Å	µ = 2.38 mm^−^^1^
α = 98.326 (1)°	*T* = 100 K
β = 94.056 (2)°	Block, brown
γ = 98.923 (1)°	0.33 × 0.16 × 0.08 mm
*V* = 1669.83 (8) Å^3^	

### Data collection

**Table:**

Bruker SMART APEXII CCD area-detector diffractometer	9594 independent reflections
Radiation source: fine-focus sealed tube	7073 reflections with *I* > 2σ(*I*)
graphite	*R*_int_ = 0.051
φ and ω scans	θ_max_ = 29.9°, θ_min_ = 2.2°
Absorption correction: multi-scan (*SADABS*; Bruker, 2005)	*h* = −14→14
*T*_min_ = 0.511, *T*_max_ = 0.838	*k* = −15→15
39493 measured reflections	*l* = −20→20

### Refinement

**Table:**

Refinement on *F*^2^	Primary atom site location: structure-invariant direct methods
Least-squares matrix: full	Secondary atom site location: difference Fourier map
*R*[*F*^2^ > 2σ(*F*^2^)] = 0.048	Hydrogen site location: inferred from neighbouring sites
*wR*(*F*^2^) = 0.133	H-atom parameters constrained
*S* = 1.06	*w* = 1/[σ^2^(*F*_o_^2^) + (0.0749*P*)^2^ + 0.6277*P*] where *P* = (*F*_o_^2^ + 2*F*_c_^2^)/3
9594 reflections	(Δ/σ)_max_ = 0.001
427 parameters	Δρ_max_ = 2.32 e Å^−^^3^
0 restraints	Δρ_min_ = −1.19 e Å^−^^3^

### Special details

**Table:**

Experimental. The crystal was placed in the cold stream of an Oxford Cyrosystems Cobra open-flow nitrogen cryostat (Cosier & Glazer, 1986) operating at 100.0 (1) K. IR spectra was recorded with a Matson 1000 FTIR spectrometer in a NaCl solution cell (0.1 mm). NMR spectra recorded in CDCl_3_ with a Bruker 400 MHz s pectrometer.
Geometry. All e.s.d.'s (except the e.s.d. in the dihedral angle between two l.s. planes) are estimated using the full covariance matrix. The cell e.s.d.'s are taken into account individually in the estimation of e.s.d.'s in distances, angles and torsion angles; correlations between e.s.d.'s in cell parameters are only used when they are defined by crystal symmetry. An approximate (isotropic) treatment of cell e.s.d.'s is used for estimating e.s.d.'s involving l.s. planes.
Refinement. Refinement of *F*^2^ against ALL reflections. The weighted *R*-factor *wR* and goodness of fit *S* are based on *F*^2^, conventional *R*-factors *R* are based on *F*, with *F* set to zero for negative *F*^2^. The threshold expression of *F*^2^ > σ(*F*^2^) is used only for calculating *R*-factors(gt) *etc*. and is not relevant to the choice of reflections for refinement. *R*-factors based on *F*^2^ are statistically about twice as large as those based on *F*, and *R*- factors based on ALL data will be even larger.

### Fractional atomic coordinates and isotropic or equivalent isotropic displacement parameters (Å^2^)

**Table:**

	*x*	*y*	*z*	*U*_iso_*/*U*_eq_	
Ru1	0.23059 (3)	0.12873 (3)	0.66692 (2)	0.01823 (9)	
Ru2	0.33724 (4)	0.23746 (3)	0.51800 (2)	0.02163 (10)	
Ru3	0.23882 (4)	0.38423 (3)	0.66627 (3)	0.02160 (10)	
As1	0.13525 (4)	0.08572 (4)	0.81172 (3)	0.01913 (11)	
O1	0.4975 (3)	0.1608 (3)	0.7762 (2)	0.0293 (8)	
O2	0.2735 (4)	−0.1180 (3)	0.5748 (3)	0.0360 (9)	
O3	−0.0494 (3)	0.0798 (3)	0.5754 (2)	0.0306 (8)	
O4	0.6137 (4)	0.2848 (3)	0.6172 (3)	0.0336 (8)	
O5	0.3994 (4)	0.0132 (4)	0.3972 (3)	0.0488 (11)	
O6	0.0618 (4)	0.1858 (4)	0.4178 (2)	0.0370 (9)	
O7	0.4120 (4)	0.4309 (4)	0.3970 (3)	0.0399 (9)	
O8	−0.0382 (4)	0.3463 (4)	0.5683 (3)	0.0538 (12)	
O9	0.1278 (4)	0.4566 (3)	0.8512 (3)	0.0351 (8)	
O10	0.5054 (4)	0.4325 (3)	0.7784 (3)	0.0379 (9)	
O11	0.3037 (4)	0.6226 (4)	0.5889 (3)	0.0493 (11)	
C1	0.2562 (4)	0.1045 (4)	0.9228 (3)	0.0223 (9)	
C2	0.3300 (5)	0.2195 (4)	0.9560 (3)	0.0280 (10)	
H2A	0.3235	0.2836	0.9231	0.034*	
C3	0.4123 (5)	0.2374 (5)	1.0376 (3)	0.0328 (11)	
H3A	0.4591	0.3144	1.0598	0.039*	
C4	0.4267 (5)	0.1425 (5)	1.0873 (3)	0.0327 (11)	
C5	0.3553 (5)	0.0296 (5)	1.0535 (4)	0.0326 (11)	
H5A	0.3651	−0.0350	1.0851	0.039*	
C6	0.2684 (5)	0.0097 (4)	0.9728 (3)	0.0264 (10)	
H6A	0.2190	−0.0666	0.9526	0.032*	
C7	0.0462 (4)	−0.0807 (4)	0.8001 (3)	0.0210 (9)	
C8	−0.0832 (4)	−0.1076 (4)	0.8174 (3)	0.0238 (9)	
H8A	−0.1272	−0.0453	0.8392	0.029*	
C9	−0.1461 (4)	−0.2265 (4)	0.8022 (3)	0.0247 (10)	
H9A	−0.2324	−0.2428	0.8140	0.030*	
C10	−0.0839 (5)	−0.3228 (4)	0.7697 (3)	0.0257 (10)	
C11	0.0470 (4)	−0.2935 (4)	0.7533 (3)	0.0260 (10)	
H11A	0.0916	−0.3555	0.7320	0.031*	
C12	0.1103 (4)	−0.1751 (4)	0.7680 (3)	0.0238 (9)	
H12A	0.1967	−0.1582	0.7563	0.029*	
C13	0.0045 (4)	0.1772 (4)	0.8591 (3)	0.0220 (9)	
C14	−0.0947 (5)	0.1967 (4)	0.7974 (3)	0.0251 (10)	
H14A	−0.0983	0.1659	0.7338	0.030*	
C15	−0.1886 (5)	0.2618 (4)	0.8300 (3)	0.0277 (10)	
H15A	−0.2551	0.2733	0.7880	0.033*	
C16	−0.1850 (5)	0.3104 (4)	0.9248 (3)	0.0269 (10)	
C17	−0.0850 (5)	0.2906 (4)	0.9860 (3)	0.0296 (11)	
H17A	−0.0805	0.3222	1.0494	0.036*	
C18	0.0087 (5)	0.2241 (4)	0.9538 (3)	0.0237 (9)	
H18A	0.0743	0.2110	0.9958	0.028*	
C19	0.5165 (5)	0.1637 (6)	1.1765 (4)	0.0462 (15)	
H19A	0.5476	0.0899	1.1846	0.069*	
H19B	0.4707	0.1888	1.2290	0.069*	
H19C	0.5885	0.2260	1.1726	0.069*	
C20	−0.1530 (5)	−0.4518 (4)	0.7531 (4)	0.0362 (12)	
H20A	−0.2420	−0.4534	0.7645	0.054*	
H20B	−0.1130	−0.4971	0.7948	0.054*	
H20C	−0.1480	−0.4873	0.6893	0.054*	
C21	−0.2861 (5)	0.3826 (5)	0.9587 (4)	0.0380 (13)	
H21A	−0.2986	0.3736	1.0226	0.057*	
H21B	−0.3661	0.3537	0.9200	0.057*	
H21C	−0.2581	0.4668	0.9551	0.057*	
C22	0.4009 (5)	0.1548 (4)	0.7333 (3)	0.0240 (10)	
C23	0.2559 (5)	−0.0277 (4)	0.6123 (3)	0.0258 (10)	
C24	0.0568 (5)	0.1036 (4)	0.6070 (3)	0.0239 (9)	
C25	0.5090 (5)	0.2675 (4)	0.5847 (3)	0.0272 (10)	
C26	0.3754 (5)	0.0942 (5)	0.4432 (4)	0.0333 (12)	
C27	0.1617 (5)	0.2062 (4)	0.4593 (3)	0.0291 (11)	
C28	0.3839 (5)	0.3601 (4)	0.4424 (3)	0.0284 (10)	
C29	0.0661 (6)	0.3529 (5)	0.6015 (4)	0.0371 (12)	
C30	0.1685 (5)	0.4274 (4)	0.7818 (4)	0.0278 (10)	
C31	0.4087 (5)	0.4051 (4)	0.7326 (3)	0.0279 (10)	
C32	0.2800 (5)	0.5349 (5)	0.6172 (4)	0.0349 (12)	

### Atomic displacement parameters (Å^2^)

**Table:**

	*U*^11^	*U*^22^	*U*^33^	*U*^12^	*U*^13^	*U*^23^
Ru1	0.02275 (18)	0.01272 (16)	0.01969 (18)	0.00001 (13)	0.00294 (14)	0.00684 (12)
Ru2	0.0286 (2)	0.01625 (17)	0.02074 (18)	0.00008 (14)	0.00498 (15)	0.00777 (13)
Ru3	0.0266 (2)	0.01339 (17)	0.0261 (2)	0.00331 (14)	0.00339 (15)	0.00697 (13)
As1	0.0225 (2)	0.0156 (2)	0.0200 (2)	0.00051 (17)	0.00296 (17)	0.00779 (16)
O1	0.0243 (18)	0.0347 (19)	0.0322 (19)	0.0051 (15)	0.0022 (15)	0.0163 (15)
O2	0.048 (2)	0.0194 (17)	0.044 (2)	0.0094 (16)	0.0120 (18)	0.0070 (15)
O3	0.0290 (19)	0.038 (2)	0.0229 (18)	0.0029 (16)	0.0010 (15)	0.0033 (15)
O4	0.031 (2)	0.037 (2)	0.035 (2)	0.0020 (16)	0.0028 (16)	0.0131 (16)
O5	0.071 (3)	0.033 (2)	0.044 (2)	0.013 (2)	0.019 (2)	0.0005 (18)
O6	0.038 (2)	0.042 (2)	0.0274 (19)	−0.0079 (18)	−0.0041 (16)	0.0119 (16)
O7	0.038 (2)	0.044 (2)	0.038 (2)	−0.0087 (18)	0.0036 (17)	0.0252 (18)
O8	0.040 (2)	0.044 (3)	0.072 (3)	0.018 (2)	−0.016 (2)	−0.010 (2)
O9	0.041 (2)	0.0279 (18)	0.038 (2)	0.0051 (16)	0.0135 (17)	0.0072 (15)
O10	0.034 (2)	0.031 (2)	0.045 (2)	−0.0017 (17)	−0.0032 (18)	0.0058 (17)
O11	0.062 (3)	0.029 (2)	0.069 (3)	0.015 (2)	0.024 (2)	0.031 (2)
C1	0.023 (2)	0.025 (2)	0.020 (2)	0.0025 (18)	0.0032 (17)	0.0098 (17)
C2	0.031 (3)	0.027 (2)	0.026 (2)	−0.002 (2)	0.003 (2)	0.0085 (19)
C3	0.029 (3)	0.038 (3)	0.028 (3)	−0.002 (2)	0.005 (2)	0.004 (2)
C4	0.021 (2)	0.053 (3)	0.026 (3)	0.014 (2)	−0.0001 (19)	0.007 (2)
C5	0.035 (3)	0.038 (3)	0.031 (3)	0.018 (2)	0.005 (2)	0.012 (2)
C6	0.028 (2)	0.028 (2)	0.026 (2)	0.007 (2)	0.0063 (19)	0.0085 (19)
C7	0.024 (2)	0.020 (2)	0.019 (2)	−0.0006 (17)	0.0005 (17)	0.0089 (16)
C8	0.027 (2)	0.021 (2)	0.026 (2)	0.0052 (18)	0.0089 (19)	0.0075 (18)
C9	0.021 (2)	0.022 (2)	0.032 (2)	−0.0026 (18)	0.0076 (19)	0.0077 (18)
C10	0.028 (2)	0.021 (2)	0.029 (2)	0.0006 (19)	0.000 (2)	0.0118 (18)
C11	0.026 (2)	0.020 (2)	0.037 (3)	0.0076 (19)	0.005 (2)	0.0145 (19)
C12	0.021 (2)	0.021 (2)	0.034 (3)	0.0072 (18)	0.0046 (19)	0.0133 (19)
C13	0.025 (2)	0.018 (2)	0.024 (2)	−0.0009 (17)	0.0044 (18)	0.0096 (17)
C14	0.031 (2)	0.026 (2)	0.018 (2)	0.0010 (19)	0.0014 (19)	0.0054 (17)
C15	0.023 (2)	0.030 (3)	0.030 (3)	0.0014 (19)	0.0019 (19)	0.010 (2)
C16	0.028 (2)	0.024 (2)	0.029 (2)	0.0022 (19)	0.003 (2)	0.0078 (19)
C17	0.039 (3)	0.027 (2)	0.024 (2)	0.005 (2)	0.006 (2)	0.0048 (19)
C18	0.027 (2)	0.026 (2)	0.021 (2)	0.0073 (19)	−0.0003 (18)	0.0102 (18)
C19	0.031 (3)	0.070 (4)	0.036 (3)	0.015 (3)	−0.006 (2)	0.003 (3)
C20	0.035 (3)	0.021 (2)	0.054 (3)	0.000 (2)	0.008 (2)	0.012 (2)
C21	0.035 (3)	0.047 (3)	0.037 (3)	0.016 (3)	0.004 (2)	0.010 (2)
C22	0.033 (3)	0.014 (2)	0.027 (2)	0.0016 (18)	0.008 (2)	0.0114 (17)
C23	0.036 (3)	0.019 (2)	0.024 (2)	0.002 (2)	0.005 (2)	0.0096 (18)
C24	0.034 (3)	0.020 (2)	0.020 (2)	0.0051 (19)	0.0072 (19)	0.0061 (17)
C25	0.036 (3)	0.025 (2)	0.024 (2)	0.007 (2)	0.008 (2)	0.0117 (19)
C26	0.045 (3)	0.027 (3)	0.028 (3)	0.003 (2)	0.009 (2)	0.007 (2)
C27	0.039 (3)	0.025 (2)	0.024 (2)	0.000 (2)	0.007 (2)	0.0107 (19)
C28	0.030 (3)	0.025 (2)	0.029 (2)	−0.002 (2)	0.000 (2)	0.0074 (19)
C29	0.043 (3)	0.023 (3)	0.044 (3)	0.013 (2)	−0.003 (3)	−0.002 (2)
C30	0.032 (3)	0.016 (2)	0.037 (3)	0.0036 (19)	0.002 (2)	0.0096 (19)
C31	0.041 (3)	0.016 (2)	0.028 (2)	0.002 (2)	0.007 (2)	0.0083 (18)
C32	0.040 (3)	0.029 (3)	0.039 (3)	0.008 (2)	0.012 (2)	0.010 (2)

### Geometric parameters (Å, °)

**Table:**

Ru1—C23	1.893 (5)	C4—C19	1.510 (7)
Ru1—C24	1.927 (5)	C5—C6	1.399 (7)
Ru1—C22	1.929 (5)	C5—H5A	0.9300
Ru1—As1	2.4629 (5)	C6—H6A	0.9300
Ru1—Ru2	2.8430 (5)	C7—C12	1.389 (6)
Ru1—Ru3	2.8745 (5)	C7—C8	1.395 (6)
Ru2—C28	1.912 (5)	C8—C9	1.381 (6)
Ru2—C26	1.925 (5)	C8—H8A	0.9300
Ru2—C27	1.933 (6)	C9—C10	1.396 (6)
Ru2—C25	1.942 (5)	C9—H9A	0.9300
Ru2—Ru3	2.8690 (5)	C10—C11	1.409 (6)
Ru3—C30	1.905 (5)	C10—C20	1.500 (7)
Ru3—C31	1.929 (6)	C11—C12	1.378 (6)
Ru3—C32	1.937 (5)	C11—H11A	0.9300
Ru3—C29	1.940 (6)	C12—H12A	0.9300
As1—C1	1.942 (5)	C13—C14	1.389 (6)
As1—C7	1.943 (4)	C13—C18	1.389 (6)
As1—C13	1.948 (4)	C14—C15	1.389 (7)
O1—C22	1.139 (6)	C14—H14A	0.9300
O2—C23	1.132 (5)	C15—C16	1.396 (7)
O3—C24	1.150 (6)	C15—H15A	0.9300
O4—C25	1.142 (6)	C16—C17	1.392 (7)
O5—C26	1.122 (6)	C16—C21	1.503 (7)
O6—C27	1.145 (6)	C17—C18	1.394 (6)
O7—C28	1.124 (6)	C17—H17A	0.9300
O8—C29	1.151 (7)	C18—H18A	0.9300
O9—C30	1.142 (6)	C19—H19A	0.9600
O10—C31	1.147 (6)	C19—H19B	0.9600
O11—C32	1.124 (6)	C19—H19C	0.9600
C1—C6	1.392 (6)	C20—H20A	0.9600
C1—C2	1.405 (6)	C20—H20B	0.9600
C2—C3	1.384 (7)	C20—H20C	0.9600
C2—H2A	0.9300	C21—H21A	0.9600
C3—C4	1.395 (8)	C21—H21B	0.9600
C3—H3A	0.9300	C21—H21C	0.9600
C4—C5	1.378 (8)		
			
C23—Ru1—C24	92.0 (2)	C1—C6—H6A	120.1
C23—Ru1—C22	89.4 (2)	C5—C6—H6A	120.1
C24—Ru1—C22	176.97 (18)	C12—C7—C8	119.1 (4)
C23—Ru1—As1	101.53 (13)	C12—C7—As1	119.2 (3)
C24—Ru1—As1	87.54 (13)	C8—C7—As1	121.6 (3)
C22—Ru1—As1	89.57 (13)	C9—C8—C7	120.1 (4)
C23—Ru1—Ru2	92.35 (13)	C9—C8—H8A	119.9
C24—Ru1—Ru2	92.87 (13)	C7—C8—H8A	119.9
C22—Ru1—Ru2	89.73 (12)	C8—C9—C10	121.9 (4)
As1—Ru1—Ru2	166.094 (19)	C8—C9—H9A	119.0
C23—Ru1—Ru3	152.51 (13)	C10—C9—H9A	119.0
C24—Ru1—Ru3	87.74 (13)	C9—C10—C11	116.9 (4)
C22—Ru1—Ru3	92.21 (13)	C9—C10—C20	121.6 (4)
As1—Ru1—Ru3	105.919 (17)	C11—C10—C20	121.4 (4)
Ru2—Ru1—Ru3	60.235 (12)	C12—C11—C10	121.5 (4)
C28—Ru2—C26	102.5 (2)	C12—C11—H11A	119.2
C28—Ru2—C27	90.7 (2)	C10—C11—H11A	119.2
C26—Ru2—C27	91.0 (2)	C11—C12—C7	120.4 (4)
C28—Ru2—C25	92.3 (2)	C11—C12—H12A	119.8
C26—Ru2—C25	90.7 (2)	C7—C12—H12A	119.8
C27—Ru2—C25	176.23 (18)	C14—C13—C18	119.2 (4)
C28—Ru2—Ru1	158.50 (15)	C14—C13—As1	119.6 (3)
C26—Ru2—Ru1	98.74 (15)	C18—C13—As1	121.2 (3)
C27—Ru2—Ru1	85.47 (13)	C13—C14—C15	120.3 (4)
C25—Ru2—Ru1	90.94 (13)	C13—C14—H14A	119.8
C28—Ru2—Ru3	98.21 (15)	C15—C14—H14A	119.8
C26—Ru2—Ru3	159.03 (15)	C14—C15—C16	121.2 (5)
C27—Ru2—Ru3	85.33 (14)	C14—C15—H15A	119.4
C25—Ru2—Ru3	91.90 (14)	C16—C15—H15A	119.4
Ru1—Ru2—Ru3	60.427 (12)	C17—C16—C15	118.0 (4)
C30—Ru3—C31	89.9 (2)	C17—C16—C21	121.5 (4)
C30—Ru3—C32	105.2 (2)	C15—C16—C21	120.5 (5)
C31—Ru3—C32	92.2 (2)	C16—C17—C18	121.1 (4)
C30—Ru3—C29	89.7 (2)	C16—C17—H17A	119.5
C31—Ru3—C29	176.4 (2)	C18—C17—H17A	119.5
C32—Ru3—C29	91.3 (2)	C13—C18—C17	120.3 (4)
C30—Ru3—Ru2	159.15 (14)	C13—C18—H18A	119.9
C31—Ru3—Ru2	86.66 (14)	C17—C18—H18A	119.9
C32—Ru3—Ru2	95.54 (15)	C4—C19—H19A	109.5
C29—Ru3—Ru2	92.39 (16)	C4—C19—H19B	109.5
C30—Ru3—Ru1	99.92 (14)	H19A—C19—H19B	109.5
C31—Ru3—Ru1	86.07 (14)	C4—C19—H19C	109.5
C32—Ru3—Ru1	154.87 (15)	H19A—C19—H19C	109.5
C29—Ru3—Ru1	90.44 (16)	H19B—C19—H19C	109.5
Ru2—Ru3—Ru1	59.337 (12)	C10—C20—H20A	109.5
C1—As1—C7	103.68 (19)	C10—C20—H20B	109.5
C1—As1—C13	101.46 (19)	H20A—C20—H20B	109.5
C7—As1—C13	101.89 (18)	C10—C20—H20C	109.5
C1—As1—Ru1	115.94 (13)	H20A—C20—H20C	109.5
C7—As1—Ru1	112.36 (12)	H20B—C20—H20C	109.5
C13—As1—Ru1	119.40 (12)	C16—C21—H21A	109.5
C6—C1—C2	119.0 (4)	C16—C21—H21B	109.5
C6—C1—As1	122.5 (4)	H21A—C21—H21B	109.5
C2—C1—As1	118.5 (3)	C16—C21—H21C	109.5
C3—C2—C1	119.9 (5)	H21A—C21—H21C	109.5
C3—C2—H2A	120.0	H21B—C21—H21C	109.5
C1—C2—H2A	120.0	O1—C22—Ru1	173.5 (4)
C2—C3—C4	121.5 (5)	O2—C23—Ru1	175.6 (4)
C2—C3—H3A	119.2	O3—C24—Ru1	173.8 (4)
C4—C3—H3A	119.2	O4—C25—Ru2	174.6 (4)
C5—C4—C3	118.1 (5)	O5—C26—Ru2	177.4 (5)
C5—C4—C19	121.1 (5)	O6—C27—Ru2	174.5 (4)
C3—C4—C19	120.8 (5)	O7—C28—Ru2	179.0 (5)
C4—C5—C6	121.6 (5)	O8—C29—Ru3	172.5 (5)
C4—C5—H5A	119.2	O9—C30—Ru3	178.1 (4)
C6—C5—H5A	119.2	O10—C31—Ru3	170.3 (4)
C1—C6—C5	119.8 (5)	O11—C32—Ru3	179.8 (5)
			
C23—Ru1—Ru2—C28	170.9 (4)	C23—Ru1—Ru3—Ru2	−4.6 (3)
C24—Ru1—Ru2—C28	78.8 (4)	C24—Ru1—Ru3—Ru2	−94.61 (12)
C22—Ru1—Ru2—C28	−99.7 (4)	C22—Ru1—Ru3—Ru2	88.42 (13)
As1—Ru1—Ru2—C28	−12.6 (4)	As1—Ru1—Ru3—Ru2	178.60 (2)
Ru3—Ru1—Ru2—C28	−7.0 (4)	C23—Ru1—As1—C1	93.0 (2)
C23—Ru1—Ru2—C26	0.4 (2)	C24—Ru1—As1—C1	−175.5 (2)
C24—Ru1—Ru2—C26	−91.7 (2)	C22—Ru1—As1—C1	3.6 (2)
C22—Ru1—Ru2—C26	89.8 (2)	Ru2—Ru1—As1—C1	−83.48 (17)
As1—Ru1—Ru2—C26	176.92 (19)	Ru3—Ru1—As1—C1	−88.53 (15)
Ru3—Ru1—Ru2—C26	−177.48 (17)	C23—Ru1—As1—C7	−26.0 (2)
C23—Ru1—Ru2—C27	90.7 (2)	C24—Ru1—As1—C7	65.6 (2)
C24—Ru1—Ru2—C27	−1.45 (19)	C22—Ru1—As1—C7	−115.3 (2)
C22—Ru1—Ru2—C27	−179.89 (19)	Ru2—Ru1—As1—C7	157.54 (16)
As1—Ru1—Ru2—C27	−92.79 (17)	Ru3—Ru1—As1—C7	152.49 (15)
Ru3—Ru1—Ru2—C27	−87.19 (15)	C23—Ru1—As1—C13	−145.2 (2)
C23—Ru1—Ru2—C25	−90.5 (2)	C24—Ru1—As1—C13	−53.6 (2)
C24—Ru1—Ru2—C25	177.40 (19)	C22—Ru1—As1—C13	125.5 (2)
C22—Ru1—Ru2—C25	−1.05 (19)	Ru2—Ru1—As1—C13	38.38 (18)
As1—Ru1—Ru2—C25	86.05 (16)	Ru3—Ru1—As1—C13	33.32 (16)
Ru3—Ru1—Ru2—C25	91.65 (14)	C7—As1—C1—C6	2.4 (4)
C23—Ru1—Ru2—Ru3	177.87 (15)	C13—As1—C1—C6	107.8 (4)
C24—Ru1—Ru2—Ru3	85.74 (13)	Ru1—As1—C1—C6	−121.3 (3)
C22—Ru1—Ru2—Ru3	−92.71 (13)	C7—As1—C1—C2	−174.9 (3)
As1—Ru1—Ru2—Ru3	−5.60 (8)	C13—As1—C1—C2	−69.5 (4)
C28—Ru2—Ru3—C30	−176.1 (5)	Ru1—As1—C1—C2	61.5 (4)
C26—Ru2—Ru3—C30	13.4 (6)	C6—C1—C2—C3	−0.6 (7)
C27—Ru2—Ru3—C30	93.9 (5)	As1—C1—C2—C3	176.8 (4)
C25—Ru2—Ru3—C30	−83.6 (5)	C1—C2—C3—C4	1.6 (7)
Ru1—Ru2—Ru3—C30	6.4 (4)	C2—C3—C4—C5	−0.7 (7)
C28—Ru2—Ru3—C31	−95.2 (2)	C2—C3—C4—C19	−179.7 (5)
C26—Ru2—Ru3—C31	94.4 (5)	C3—C4—C5—C6	−1.3 (7)
C27—Ru2—Ru3—C31	174.85 (19)	C19—C4—C5—C6	177.7 (5)
C25—Ru2—Ru3—C31	−2.59 (18)	C2—C1—C6—C5	−1.3 (6)
Ru1—Ru2—Ru3—C31	87.41 (13)	As1—C1—C6—C5	−178.6 (3)
C28—Ru2—Ru3—C32	−3.2 (2)	C4—C5—C6—C1	2.3 (7)
C26—Ru2—Ru3—C32	−173.7 (5)	C1—As1—C7—C12	−74.5 (4)
C27—Ru2—Ru3—C32	−93.2 (2)	C13—As1—C7—C12	−179.6 (4)
C25—Ru2—Ru3—C32	89.3 (2)	Ru1—As1—C7—C12	51.4 (4)
Ru1—Ru2—Ru3—C32	179.35 (18)	C1—As1—C7—C8	109.7 (4)
C28—Ru2—Ru3—C29	88.3 (2)	C13—As1—C7—C8	4.6 (4)
C26—Ru2—Ru3—C29	−82.1 (5)	Ru1—As1—C7—C8	−124.4 (3)
C27—Ru2—Ru3—C29	−1.7 (2)	C12—C7—C8—C9	−0.3 (7)
C25—Ru2—Ru3—C29	−179.1 (2)	As1—C7—C8—C9	175.5 (3)
Ru1—Ru2—Ru3—C29	−89.09 (17)	C7—C8—C9—C10	0.1 (7)
C28—Ru2—Ru3—Ru1	177.43 (15)	C8—C9—C10—C11	0.2 (7)
C26—Ru2—Ru3—Ru1	7.0 (5)	C8—C9—C10—C20	−179.8 (5)
C27—Ru2—Ru3—Ru1	87.44 (13)	C9—C10—C11—C12	−0.4 (7)
C25—Ru2—Ru3—Ru1	−90.00 (13)	C20—C10—C11—C12	179.6 (5)
C23—Ru1—Ru3—C30	177.7 (4)	C10—C11—C12—C7	0.3 (7)
C24—Ru1—Ru3—C30	87.7 (2)	C8—C7—C12—C11	0.1 (7)
C22—Ru1—Ru3—C30	−89.3 (2)	As1—C7—C12—C11	−175.8 (4)
As1—Ru1—Ru3—C30	0.93 (16)	C1—As1—C13—C14	173.4 (4)
Ru2—Ru1—Ru3—C30	−177.67 (16)	C7—As1—C13—C14	−79.8 (4)
C23—Ru1—Ru3—C31	−93.0 (4)	Ru1—As1—C13—C14	44.6 (4)
C24—Ru1—Ru3—C31	176.95 (18)	C1—As1—C13—C18	−6.3 (4)
C22—Ru1—Ru3—C31	−0.02 (18)	C7—As1—C13—C18	100.5 (4)
As1—Ru1—Ru3—C31	90.16 (14)	Ru1—As1—C13—C18	−135.1 (3)
Ru2—Ru1—Ru3—C31	−88.44 (14)	C18—C13—C14—C15	−0.2 (7)
C23—Ru1—Ru3—C32	−6.1 (5)	As1—C13—C14—C15	−179.9 (3)
C24—Ru1—Ru3—C32	−96.1 (4)	C13—C14—C15—C16	0.8 (7)
C22—Ru1—Ru3—C32	86.9 (4)	C14—C15—C16—C17	−0.6 (7)
As1—Ru1—Ru3—C32	177.1 (4)	C14—C15—C16—C21	178.9 (5)
Ru2—Ru1—Ru3—C32	−1.5 (4)	C15—C16—C17—C18	−0.1 (7)
C23—Ru1—Ru3—C29	87.9 (4)	C21—C16—C17—C18	−179.6 (5)
C24—Ru1—Ru3—C29	−2.1 (2)	C14—C13—C18—C17	−0.5 (7)
C22—Ru1—Ru3—C29	−179.1 (2)	As1—C13—C18—C17	179.2 (3)
As1—Ru1—Ru3—C29	−88.88 (17)	C16—C17—C18—C13	0.7 (7)
Ru2—Ru1—Ru3—C29	92.52 (17)		

### Hydrogen-bond geometry (Å, °)

**Table:**

*D*—H···*A*	*D*—H	H···*A*	*D*···*A*	*D*—H···*A*
C14—H14A···O3	0.93	2.48	3.385 (5)	165
